# Supplementary material for: Nature and type of patient-reported safety incidents at a tertiary hospital in South Africa during the COVID-19 period (2018–2021)- A retrospective review
Source: PLoS One. 2023 Nov 9;18(11):e0293933. doi: 10.1371/journal.pone.0293933 (PMC10635523; doi:10.1371/journal.pone.0293933)
Supplement: Attachment — Submitted filename: reviwer comments.docx [file pone.0293933.s002.docx]

The Editor Plos One

Reviewers Comments

I wish to acknowledge the reviewers for their positive comments on the manuscript. It is really appreciated, noting the limitations when reporting on patient safety incidents in an environment that is still not very conducive to such. We acknowledge the limitations of this study and attempted to respond to the best of our ability to the reviewers.

Reviewer ⌗ 1:

| 1. | The PSIs were reported using the standardized forms of the South African National Guidelines for Patient Safety Reporting. These forms should be described, as well as the way of reporting (paper forms or electronic system) | We have added a description under the section Data Collection Tool and Procedures Line 83-103 |
| --- | --- | --- |
| 2. | the distribution of reporting by medical doctors and nursing staff should be explained | The majority of the patient safety events were reported by nursing staff. |
| 3. | the education of the hospital staff for the PSI reporting should be opened. Is the improved reporting from 2018 to 2021 explained e.g. by improved training of the staff? | The improved reporting is a combination of training as well as more focussed attention by the National and Provincial Health Departments on the completion and reportion of adverse events in response to the increased medical litigation. (Line 315-317) |
| 4. | - were the patients of the hospital also able to file PSIs? | Patients usually report any complaints or adverse events through the complaints mechanism at the hospital. |

Reviewer ⌗ 2:

| 1. | The investigators should refocus the manuscript on the incidents that are less prone to reporting bias | Reviewers point acknowledged. It was our intention to provide the prevalence as reported, with the limitations. We will write a separate manuscript with focussed patient safety incidents. |
| --- | --- | --- |
| 2. | The demographics (Table 2) are of interest and correlations with severity of incidents will be informative | We thank reviewer for the comments. It is our intention to submit. Second manuscript looking at bivariate and multivariate analysis using demographic variables |
| 3. | more information are required about the data source: what requires reporting, how is reporting done, analysis and verifying data when reported, root cause analysis, strategies to minimize reporting bias and encourage reporting | Modifications made between line 87 and 109 |
|  | Past tense & future tense used in the same paragraph | Corrected |
|  | There is no reflection on the South African context. | Very few previous studies conducted in South Africa. Line 301-304 quote a previous study in the same institution. |
|  |  |  |

-
