# Supplementary material for: Nature and type of patient-reported safety incidents at a tertiary hospital in South Africa during the COVID-19 period (2018–2021)- A retrospective review
Source: PLoS One. 2023 Nov 9;18(11):e0293933. doi: 10.1371/journal.pone.0293933 (PMC10635523; doi:10.1371/journal.pone.0293933)
Supplement: Attachment — Submitted filename: reviwer comments2.docx [file pone.0293933.s003.docx]

The Editor Plos One

Reviewers Comments

Thank you for the opportunity to resubmit revisions to the manuscript. We acknowledge the effort of the reviewers. We apologise to the reviewer for any offence that many have been caused by our dismissive resposes. We have tried to align our responses and make the necessary changes.

Reviewer ⌗ 2:

| 1. | The investigators should refocus the manuscript on the incidents that are less prone to reporting bias | We have focussed on five main adverse events |
| --- | --- | --- |
| 2. | The demographics (Table 2) are of interest and correlations with severity of incidents will be informative | Table 2 revised |
|  | There is no reflection on the South African context. | We have shortened the discussion.We have included the study suggested by the reviewer. |
|  |  |  |

-
